# Fine-grained urban blue-green-gray landscape dataset for 36 Chinese cities based on deep learning network

**DOI:** 10.1038/s41597-023-02844-2

**Published:** 2024-03-04

**Authors:** Zhiyu Xu, Shuqing Zhao

**Affiliations:** 1 College of Urban and Environmental Sciences, Peking University, Beijing, 100871 China; 2 College of Ecology and the Environment, Hainan University, Haikou, 570228 China

**Keywords:** Environmental social sciences, Geography, Ecology

## Abstract

Detailed and accurate urban landscape mapping, especially for urban blue-green-gray (UBGG) continuum, is the fundamental first step to understanding human–nature coupled urban systems. Nevertheless, the intricate spatial heterogeneity of urban landscapes within cities and across urban agglomerations presents challenges for large-scale and fine-grained mapping. In this study, we generated a 3 m high-resolution UBGG landscape dataset (UBGG-3m) for 36 Chinese metropolises using a transferable multi-scale high-resolution convolutional neural network and 336 Planet images. To train the network for generalization, we also created a large-volume UBGG landscape sample dataset (UBGGset) covering 2,272 km^2^ of urban landscape samples at 3 m resolution. The classification results for five cities across diverse geographic regions substantiate the superior accuracy of UBGG-3m in both visual interpretation and quantitative evaluation (with an overall accuracy of 91.2% and FWIoU of 83.9%). Comparative analyses with existing datasets underscore the UBGG-3m’s great capability to depict urban landscape heterogeneity, providing a wealth of new data and valuable insights into the complex and dynamic urban environments in Chinese metropolises.

## Background & Summary

Urban Landscapes are complex and dynamic geographic phenomena that are shaped by natural and human forces^1,2^. These landscapes are comprised of various components, including urban green space (UGS), urban blue space (UBS), and urban impervious surfaces (UIS), which together form the basic units of complex urban landscape configurations^3,4^. UGS generally refers to vegetated land in urban areas^5^, such as parks, gardens, trees, and grasses, which play a crucial role in upholding urban ecosystem equilibrium. UBS pertains to water features within urban locales, encompassing rivers, lakes, wetlands, reservoirs, ponds, and artificial water structures^3^. UIS, commonly referred to as “urban gray space”, are impervious surface features in cities caused by man-made land use activities, such as building roofs, asphalt or concrete roads^6^. UGS and UBS provide multiple ecological and social benefits, including climate regulation, water and air purification, biodiversity conservation, carbon sequestration, recreational opportunities, and aesthetic enhancement^3,7,8^. However, UIS may engender negative impacts, such as the urban heat island (UHI) effect, air quality degradation, and stormwater runoff increases^6,9^. Therefore, a balanced approach to urban landscape construction necessitates the integration of all three space types, with a focus on increasing the quantity and quality of UGS and UBS, while also minimizing the negative impacts of UIS, which can lead to a more sustainable and livable urban environment^10^. In the context of global urbanization, there is a pressing need for a more precise perception of the urban landscape structure^11^. Nonetheless, urban blue-green-gray (UBGG) landscapes are strikingly heterogeneous in terms of space, structure and function, as they are the outcome of dynamic interactions between biophysical and socio-economic processes occurring at multiple spatial scales^2,12^. Yet, detailed and accurate UBGG landscape mapping is the fundamental first step to understanding human–nature coupled urban systems. Therefore, it is necessary to open the “closed box” of urban landscape structure and quantify the subtle heterogeneity of the built and natural components within the metropolis^1,13^.

With the increased demand for higher resolution urban products, urban mapping products have made tremendous progress toward finer scales in the last decades^11,14,15^. It can be attributed to the availability and accessibility of very high resolution (VHR) satellite data and the support of computing platforms with substantial computing power, such as Google Earth Engine (GEE)^11^. Notably, VHR imagery, with resolutions as fine as 1–3 m/pixel, has emerged as an invaluable asset for revealing urban landscapes at an increasingly detailed level of granularity, offering a comprehensive view of the ground. Moreover, Deep Learning (DL) techniques have emerged as a powerful tool for VHR urban landscape mapping, revolutionizing the field of intelligent classification research in the 21st century^16–18^. Establishing fine-scale urban datasets for landscape/landcover interpretation and deep learning-based research has become a hot research topic in recent years^15,17,19^. However, present urban landscape datasets typically focused on individual landscapes (e.g., UGS or UBS)^20,21^ or limited spatial extents (usually covering several cities/provinces)^22^. Some scholars focused on UGS extraction to accurately digitally twin UGS at a fine scale^5,21,23^, such as Brandt *et al*.^23^ utilized VHR satellite images covering more than 1.3 million km^2^ in West African Sahara and Sahel, detecting more than 1.8 billion individual trees in areas previously regarded as barely covered by trees. Similarly, Shi *et al*.^5^ generated 1-meter UGS maps for 31 major cities in China using Google Earth images. Some scholars focused on UBS extraction to address the obstacles posed by the confusion of water with heavy shadows in VHR images^20,24,25^, like Chen *et al*.^25^ proposed an open water detection method in urban areas using VHR imagery, successfully identifying various types of water bodies. Likewise, Li *et al*.^20^ proposed the water index-driven deep fully convolutional network (WIDFCN), showcasing robustness to different shadows types and achieving high-performance water extraction in 12 test sites worldwide. In addition, in the field of UIS extraction, scholars have also achieved notable outcomes in the application of DL to urban building and road extraction from VHR images^26–28^. For example, Guo *et al*.^28^ devised a coarse-to-fine boundary refinement network for building footprints extraction from VHR images. Nevertheless, a limitation persists in the mapping of single landscapes or confining analyses to limited geographical extents, failing to offer a comprehensive understanding of the highly heterogeneous interactions between human and natural elements^1,22^.

Establishing an effective automatic DL model for fine-grained and large-scale UBGG dataset is a challenging frontier in high-resolution urban landscape mapping. However, the pursuit of such datasets comes with its own set of challenges, stemming from VHR image acquisition, manual annotation, and the intrinsic heterogeneity of urban landscapes. First, the paramount significance of VHR imagery in capturing intricate urban landscape details is countered by its inherent costliness and the complexities associated with its acquisition^15,29^. Although Google Images has been used for some large-scale research, its restricted geographic and temporal coverage, limited visible spectrum bands, as well as varying image quality are also inevitable drawbacks^14^. Second, training a UBGG network with large-scale applications and high generalization capability relies on a large-volume sample dataset^21^, which poses a major challenge for nationwide landscape mapping due to the enormous dataset, laborious annotation, and cumbersome process involved. Although some studies have proposed innovative techniques employing biophysical indices or existing coarse-resolution products in conjunction with self-supervised mechanisms to generate training labels automatically^20^, the label noise of resolution mismatch of spatial resolution and the true accuracy of labels require further scrutiny. Reliable training labels are crucial to achieving accurate fine-scale landscape mapping results but still insufficient. Third, the striking heterogeneity characterizing the UBGG landscape at both intra- and inter-city levels and across various spatial scales presents significant impediments to effectively mining multi-scale features^1,2^. The variability of urban landscapes across geographic locations and climatic zones, such as plant type, water quality, building structure, and color, poses significant challenges^30^. Additionally, mining multi-scale features from UBGG landscapes presents substantial obstacles. Fine-scale features, encompassing spectral colors, geometrical sizes, and textural shapes, primarily manifest in the network’s shallow layers but are often confused and invalidated at deeper levels^26^. Conversely, coarse-scale features, such as global spatial context, are obtained from the deep layer but struggle to be effectively expressed^31^.

China has undergone rapid development and urbanization in recent decades^32^, becoming the world’s second-largest economy. In light of this remarkable growth, a comprehensive mapping survey of large-scale and fine-grained landscapes assumes immense significance, fostering an in-depth comprehension of urban environment, facilitating effective urban landscape management, and illuminating future development trajectories^33^. Consequently, this study endeavors to develop a transferable multi-scale high-resolution convolutional neural network to generate a 3-meter resolution UBGG landscape dataset, utilizing Planet images in 36 Chinese metropolises. Rigorous validation processes, including visual interpretation and quantitative evaluations, were employed to assess the credibility and efficacy of the UBGG-3m dataset, further augmented by comparisons with existing products. This dataset will enhance our understanding of fine-scale landscape distribution patterns in Chinese metropolises, provide a deeper understanding of integrated human-nature systems from an ecological perspective, and contribute to better urban landscape management as well as sustainable urban development planning^1,12,29^.

## Methods

### Data collection and pre-processing

**Fig. 1:**
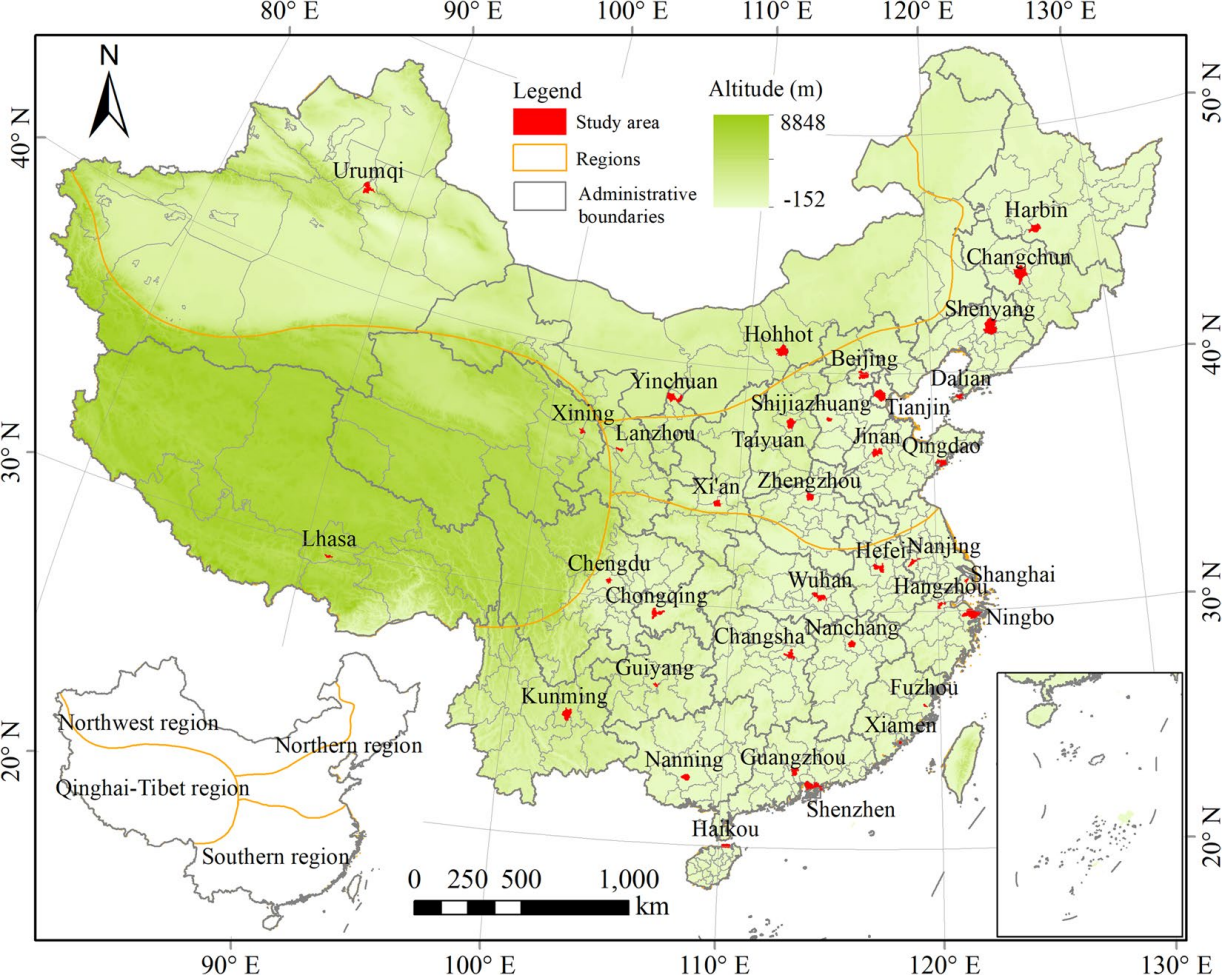
Spatial distribution of the 36 study cities within the four geographic regions in China.

To supplement the lack of large-scale, fine-grained landscape datasets, this study used Planet multispectral satellite images and ancillary data to create UBGG-3m dataset. The dataset encompasses 36 Chinese metropolises, including urban areas of 22 provincial capitals, 5 autonomous region capitals, 4 municipalities directly under the central government, and 5 municipalities with independent planning status (Fig. 1). To account for China’s vast territorial expanse and the heterogeneity of its landforms, the 36 metropolises were divided into four major geographic regions^34^: the northern region (Harbin, Changchun, Shenyang, Dalian, Beijing, Tianjin, Shijiazhuang, Taiyuan, Lanzhou, Qingdao, Jinan, Zhengzhou, and Xi’an), the southern region (Shanghai, Nanjing, Hangzhou, Hefei, Ningbo, Wuhan, Changsha, Nanchang, Chengdu, Chongqing, Guiyang, Kunming, Nanning, Fuzhou, Xiamen, Guangzhou, Shenzhen, and Haikou), the northwest region (Hohhot, Yinchuan, and Urumqi), and the Qinghai-Tibet region (Xining and Lhasa).

Planet multispectral satellite images with a spatial resolution of 3 meters provide an important data source for capturing the detailed characteristics of urban landscapes (https://www.planet.com/explorer/). Planet, with the largest commercial Earth observation satellite constellation ever built, operates over 200 small satellites in near-Earth orbit^35^. These satellites provide meter and sub-meter spatial resolution images, allowing for an unprecedented global repeat observation frequency of once a week. This frequency and resolution enable the capture and analysis of UBGG landscapes in unprecedented detail, providing insights into the morphology and dynamics of the urban landscape at an unprecedented scale. In addition, the Planet multispectral imagery comprises four bands, with the near-infrared band being particularly adept at capturing vegetation growth information, thereby augmenting the accuracy of UGS type classification. A total of 336 clear and non-cloudy images in summer of 2020 (June to October) were downloaded (Table 1). In cases where cloud cover obscured images of the study area in 2020, cloud-free images from the summer of 2021 served as suitable replacements. The Planet satellite images were preprocessed utilizing geometrical correction, image mosaic, color stretching, band combination, and projection transformation. Finally, we obtained standard false color images covering 36 metropolitan urban areas in China, where trees and grass are shown as dark and bright red, which can be better distinguished from UBS and UIS.

**Table 1:** Information of the Planet satellite images used in this study.

	Province	City	Acquisition images date	Number of images
Provincial capital (22)	Anhui	Hefei	20210910	12
	Fujian	Fuzhou	20200722	2
	Gansu	Lanzhou	20200728, 20200802	9
	Guangdong	Guangzhou	20201011, 20201012	5
	Guizhou	Guiyang	20210606	3
	Hainan	Haikou	20200729, 20200824	4
	Hebei	Shijiazhuang	20200916	5
	Heilongjiang	Harbin	20200721, 20200821	8
	Henan	Zhengzhou	20200829	9
	Hubei	Wuhan	20210722, 20210724	10
	Hunan	Changsha	20200815, 20200816	10
	Jiangsu	Nanjing	20210730	13
	Jiangxi	Nanchang	20210722	7
	Jilin	Changchun	20200721, 20200726	15
	Liaoning	Shenyang	20210828, 20210829	15
	Qinghai	Xining	20200719, 20200726	5
	Shaanxi	Xi’an	20200702, 20200707	8
	Shandong	Jinan	20200903, 20200930	8
	Shanxi	Taiyuan	20210730	9
	Sichuan	Chengdu	20200727, 20200728	8
	Yunnan	Kunming	20210912	16
	Zhejiang	Hangzhou	20200816	3
Autonomous region (5)	Guangxi	Nanning	20210606	9
	Inner Mongolia	Hohhot	20200914, 20200919	8
	Ningxia	Yinchuan	20210704	10
	Tibet	Lhasa	20210724	5
	Xinjiang Uygur	Urumqi	20200802, 20200804, 20200805	10
Municipality (4)		Beijing	20200809, 20200811	11
		Chongqing	20200826	18
		Shanghai	20210623, 20210714, 20210715	27
		Tianjin	20200828, 20200903	9
Municipalities with independent planning status (5)	Fujian	Xiamen	20210722	1
	Guangdong	Shenzhen	20201026, 20201027	14
	Liaoning	Dalian	20200708, 20200725	7
	Shandong	Qingdao	20210619	8
	Zhejiang	Ningbo	20210714, 20210715	15

The boundaries of 36 metropolises were defined according to the administrative boundaries, which were obtained from the Resource and Environment Science and Data Center (https://www.resdc.cn). Nonetheless, administrative boundaries cannot distinguish between urban and rural areas, leading to potential misclassification of urban grassland and farmland, due to their similar physical features but distinct economic attributes. To address this challenge and improve classification accuracy, we integrated the 2018 China Urban Boundary (CUB) data^4^ into our classification process. The CUB data was meticulously extracted through a human–computer digitalization process from China’s Land Use/cover Dataset (CLUD), derived from Landsat images. Notably, the CUB data is known for its high accuracy in urban boundary detection, with an overall accuracy rate exceeding 92.65% from 2000 to 2018^4^. Specifically, we focused on reclassifying areas outside the urban boundaries, ensuring that urban grasslands located outside these boundaries were accurately reclassified as farmland.

To comprehensively assess the reliability and precision of the UBGG-3m dataset, we collected several established and widely utilized land cover datasets, as well as two high-resolution urban green space datasets for comparison and validation. Specifically, the land cover datasets include the 30 m GlobeLand30 in 2020^36^, the 10 m Esri land cover in 2020^37^, the 10 m ESA World Cover in 2020^38^, the 1 m national-scale land-cover map (SinoLC-1m)^14^. To ensure consistency with our classification system, the four land cover products were reclassified into UGS (trees, grassland and farmland), UBS, and UIS. The other two high-resolution urban green space datasets include the 2 m Urban Tree Cover dataset (UTC-2m)^21^, and 1 m Urban Green Space (UGS-1m)^5^.

### Technical framework

**Fig. 2:**
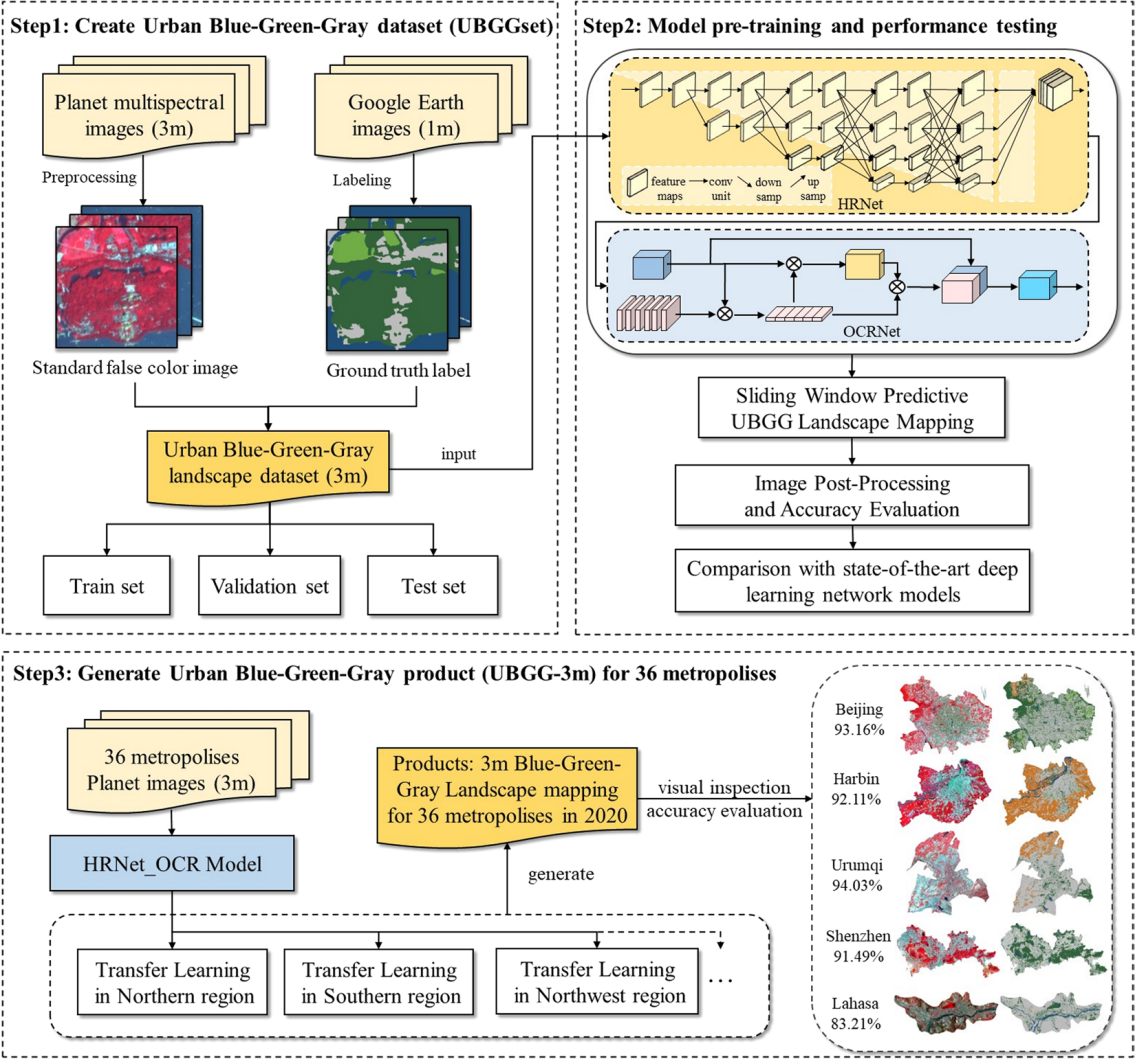
Workflow for generating the Urban Blue-Green-Gray landscape product (UBGG-3m) using high-resolution Planet satellite images.

The workflow for generating the UBGG-3m dataset mainly includes three phases, as depicted in Fig. 2. Firstly, UBGGset for typical Chinese cities was created for training, validation, and testing of the deep learning model. Secondly, the novel deep learning model was pre-trained on the UBGGset and tested in Beijing to compare its performance against state-of-the-art deep learning network models. Finally, the transfer training was utilized to strengthen the pre-trained model to adapt to diverse landscape characteristics in different geographic regions and generated UBGG-3m of 36 metropolitan areas in China. Thorough visual inspection and quantitative accuracy validation were conducted to ensure the reliability and credibility of the UBGG-3m dataset.

#### UBGG landscapes sample dataset

**Fig. 3:**
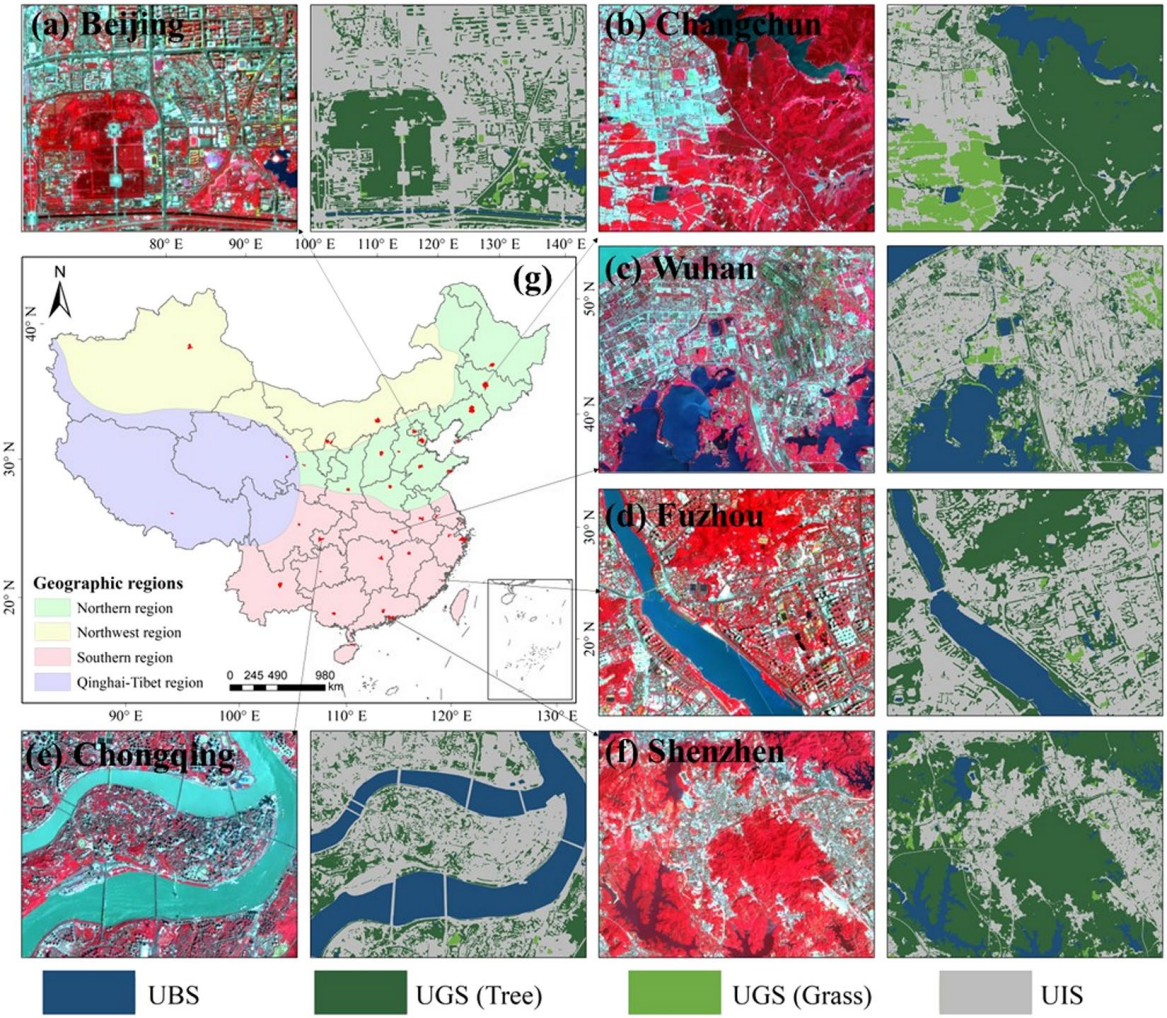
Examples of Urban Blue-Green-Gray sample dataset (UBGGset) for six cities: (**a**) Beijing, (**b**) Changchun, (**c**) Wuhan, (**d**) Fuzhou, (**e**) Chongqing, and (**f**) Shenzhen. The UBGGset is constructed with co-registered pairs of 3 m Planet satellite images from © Planet 2020 (left) and fine-annotated urban landscapes labeled (right).

Accurate and reliable training tags are critical to the accuracy of fine-scale urban landscape mapping^14,20^. Current UBGG studies are deficient in standard datasets, so we first created a large-volume UBGG landscape sample dataset (UBGGset) applicable to urban areas in China. The classification system includes the UBS, UGS, and UIS landscapes in the city. UBS comprises all water bodies, including rivers, lakes, and seas, as well as reservoirs and ponds, while UGS is further divided into tree, grass, and farmland. The others are classified as UIS, including buildings, traffic roads, squares, and other impervious surfaces. In addition, shaded and bare land is also classified as UIS. UBGGset was constructed with co-registered pairs of 3 m Planet images and fine-annotated urban landscapes labeled on 1 m Google Earth images. The visual interpretation process of the UBGGset landscapes was done by the mapping team and further validated by field surveys. Moreover, UBGGset covers 4 major geographic regions and 15 typical cities (Beijing, Harbin, Changchun, Hefei, Wuhan, Changsha, Xi’an, Chengdu, Chongqing, Guizhou, Fuzhou, Shenzhen, Hohhot, Lanzhou, and Lhasa), covering an urban area of about 2,272 km^2^, which enriches the urban landscape standard datasets and facilitates the large-scale application of deep network. Examples of UBGGset for six cities are shown in Fig. 3. After that, 50852 training images and 12712 validation images (Length 256 × width 256) were obtained by sliding window clipping and data enhancement (horizontal, vertical, and diagonal flip).

#### HRNet-OCR network architecture

**Fig. 4:**
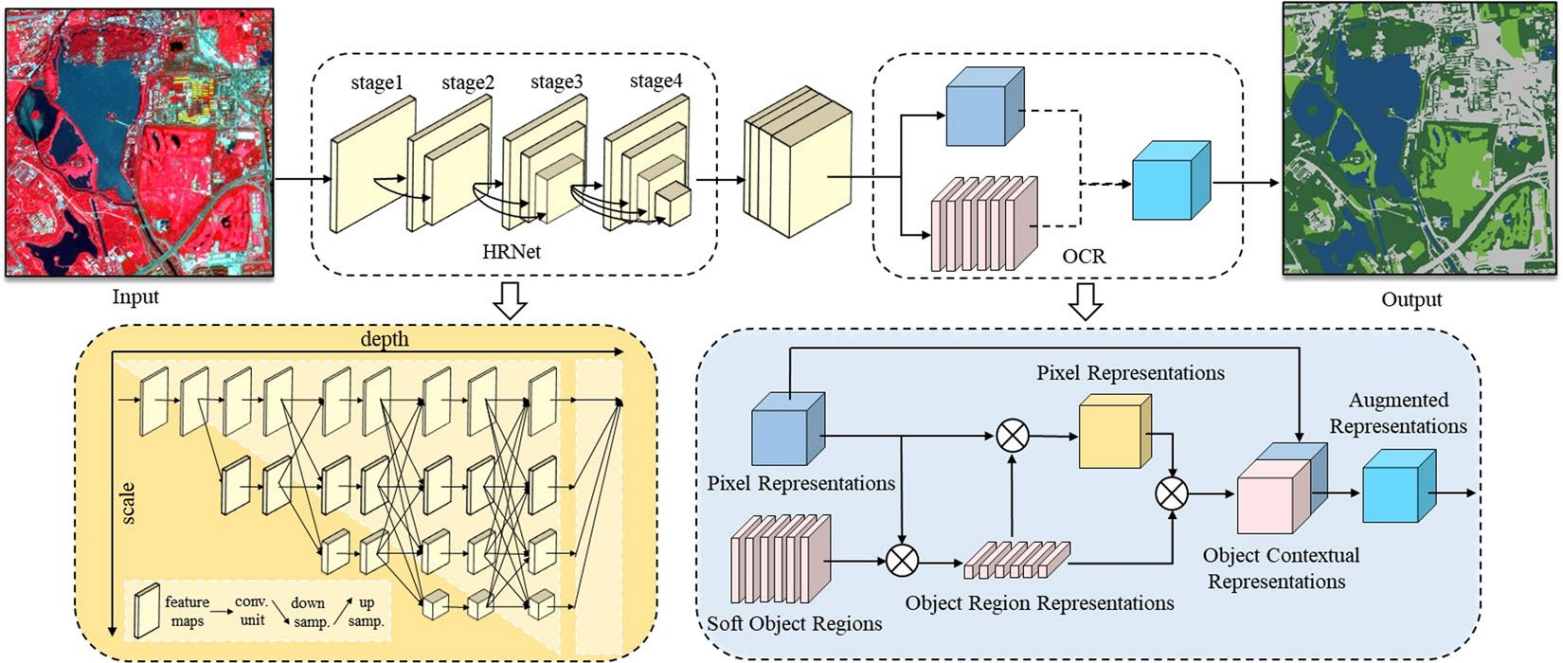
The architecture of the HRNet-OCR. The yellow box shows the structure of HRNet^39^, and the blue box shows the structure of OCR module^31^.

The HRNet-OCR network architecture (Fig. 4), constituting the core of the deep learning model, was designed to tackle the challenges posed by the multi-scale information extraction and the inadequacy of contextual information in VHR images. Leveraging the High-Resolution Network (HRNet)^39^ as the backbone network, HRNet-OCR effectively harnessed multi-scale feature learning and exploited four multi-branch parallel convolutions to generate high-to-low-resolution feature maps^40^. Meanwhile, multi-scale information branches are sufficiently linked to enable the seamless flow of information and enhancing semantic richness and spatial accuracy. Furthermore, this structure can effectively avoid the loss caused by the recovery of high-resolution features from low-resolution features, thereby preserving the image’s high-resolution features throughout the process. To overcome the problem of inadequate contextual information, we also integrated the Object-Contextual Representations (OCR) module^31^ into model. The OCR module is designed to capture global context information and integrate it with local features to enhance the model’s ability to recognize and distinguish objects in VHR images. It also includes a feature fusion component that combines the extracted features with the original feature map. This process enables the model to integrate both local and global context information, thereby improving its capability to recognize objects in complex scenes with multiple objects and occlusions.

The specific training steps are as follows:

Firstly, we input the train and validation dataset into the HRNet for multi-scale feature learning and then obtained a coarse segmentation map from the softmax layer.

Secondly, we computed the object regions from the coarse segmentation map from HRNet by aggregating the representations of all pixels in the *N*^th^ landscape object.

1 $${f}_{N}=\sum _{i\in \tau }{\widetilde{m}}_{Ni}{x}_{i}$$

Where *N* is the number of landscape categories, *f*_*N*_ represents the *N* landscape object. *x*_*i*_ denotes the pixel *i* representation. ${\widetilde{m}}_{Ni}$ refers to the normalized degree for pixel *i* in the *N*^th^ landscape object.

Thirdly, we calculated the relationship between pixels and the corresponding landscape object as below:

2 $${w}_{iN}=\frac{{e}^{N({x}_{i},{f}_{N})}}{{\sum }_{j=1}^{N}{e}^{N({x}_{i},{f}_{N})}}$$

Where *W*_*iN*_ means the relationship between *x*_*i*_ and *f*_*N*_. The transformation function *N(x, f)* is referenced in literature^31^.

Lastly, the final pixel representation *z*_*i*_ was obtained by computing the combination of the original representation *x*_*i*_ and the object background representation *y*_*i*_ using the transformation function g(*·*)^31^.

3 $${z}_{i}=g\left({\left[{x}_{i}^{{\rm{T}}}{y}_{i}^{{\rm{T}}}\right]}^{{\rm{T}}}\right)$$

4 $${y}_{i}=\rho \left(\mathop{\sum }\limits_{N=1}^{4}{w}_{iN}\delta \left({f}_{N}\right)\right)$$

Where *z*_*i*_ is the augmented pixel representation; *y*_*i*_ is the object contextual representation.

#### Transfer learning

To enable the model’s effective performance across large-scale applications, the study employed a transfer learning technique^15^. Influenced by natural factors (e.g., vegetation type, spectral diversity of water bodies, impermeable styles) and external factors (e.g., solar altitude angle, image quality)^1^, satellite images collected from different regions could exhibit inconsistent data distribution^15^. Therefore, a model trained on one region dataset cannot be applied effectively to images of another region. To overcome this challenge, transfer learning was employed, where a pre-trained model was used as a starting point for a new task in different geographic regions. Specifically, we first trained a model in the Northen geographic region to obtain a pre-trained model and further fine-tuned it through adversarial training by adding samples from the next region to the pre-trained model for parameter initialization and feature extraction (as shown in Fig. 2). This process was repeated for each geographic region. For large-scale applications, transfer learning can improve computing efficiency and model generalization compared to starting from scratch on a small sample dataset.

#### Post-processing

In the post-processing stage of image classification, three essential techniques were implemented to enhance the accuracy of the results. The sliding window prediction method^27^ was employed to effectively address the issue of insufficient image edge information, mitigating the impact of mosaic traces. Test enhancement techniques, involving horizontal, vertical, and diagonal flipping, were used to improve classification accuracy and reliability by averaging test image enhancements. Lastly, morphological post-processing, facilitated by the “skimage” package in Python, removed small incorrect patches and filled tiny holes, ensuring precise and accurate classification results.

#### Accuracy assessment

To evaluate the accuracy and quality of the proposed UBGG-3m dataset, comprehensive assessments were conducted at the pixel level. The widely used assessment metrics were used to evaluate classification accuracy for each landscape pixel, including Precision, Recall, overall accuracy (OA), F1-score (F1), intersection over union (IoU), and frequency weighted intersection over union (FWIoU). The calculation equations for the metrics are shown in Table 2.

**Table 2:** Assessment metrics.

Assessment metrics	Formula
Precision	$Precision=\frac{TP}{TP+FP}$
Recall	$Recall=\frac{TP}{TP+FN}$
F1-Score	$F1=\frac{2\times Precision\times Recall}{Precision+Recall}$
Overall accuracy	$OA=\mathop{\sum }\limits_{i=1}^{n}\frac{{x}_{ii}}{N}$
IoU	$IoU=\frac{TP}{TP+FP+FN}$
FWIoU	$FWIoU=\frac{1}{{\sum }_{i=0}^{K}{\sum }_{j=0}^{K}{x}_{ij}}\mathop{\sum }\limits_{i=0}^{K}\frac{{\sum }_{j=0}^{K}{x}_{ij}{x}_{ii}}{{\sum }_{j=0}^{K}{x}_{ij}+{\sum }_{j=0}^{K}{x}_{ji}-{x}_{ii}}$

Where *TP*, *TN*, *FP* and *FN* represent true positive, true negative, false positive and false negative; *N* is the total pixel number of the image. *K* is the number of categories. *x*_*ii*_ represents the pixel number of the category *i* that was correctly classified. *x*_*ij*_ represents the pixel number of the category *i* that are wrongly divided into category *j*.

#### Experimental parameters

The whole experimental process was completed on the High-performance Computing Platform of Peking University, employing the Pytorch deep learning framework with GPU acceleration from NVIDIA Tesla P100. During the training process, the batch size was 32, and the initial learning rate was 0.0001. The learning rate was adjusted by simulated annealing to avoid the possibility of the gradient descent algorithm falling into local minima, with a minimum learning rate of 1e-5. The Adam optimizer was selected for loss-value optimization with a weight decay factor of 0.001. We set the number of iteration epochs to 120 and selected the optimal model parameters corresponding to the rounds with the highest accuracy in the training and validation. The loss function is utilized to calculate the difference between the predicted and true values and update the network model parameters by error backpropagation. Here, we used the combined loss function of Soft Cross Entropy Loss (CE) and Dice Loss (DL)^5^, which can more effectively solve the category imbalance problem and enhance the model generalization. The calculation formula is as follows:

5 $$Loss={w}_{CE}Los{s}_{CE}+{w}_{DL}Los{s}_{DL}$$

6 $$Los{s}_{CE}=\frac{1}{N}\sum -[{y}_{i}\cdot log({p}_{i})+(1-{y}_{i})\cdot log(1-{p}_{i})$$

7 $${L}_{DL}=1-\frac{\sum _{i}| {p}_{i}\cap {y}_{i}| }{\sum _{i}(| {p}_{i}| +| {y}_{i}| )}$$

where *y*_*i*_ is the prediction of urban landscapes of the network. *p*_*i*_ is the truth of urban landscapes from label images. The weights of *w*_*CE*_ and *w*_*DL*_ are 0.5.

## Data Records

The UBGG dataset^41^ provides easily access and leverage to researchers and analysts, which is stored in the following Zenodo repository (10.5281/zenodo.8352777). The UBGG dataset consists of two main components:

1. **UBGG-3m:** the fine-grained UBGG map of 36 metropolises in China. The UBGG-3m dataset captures the intricate urban landscape features with remarkable precision, providing a detailed representation at an impressive 3-meter resolution. The classification maps for all 36 Chinese metropolises were showcased in Fig. 5. Researchers can delve into the nuances of the UBGG continuum, gaining invaluable insights into the interplay between the blue, green, and gray elements of urban environments in each metropolis.

**Fig. 5:**
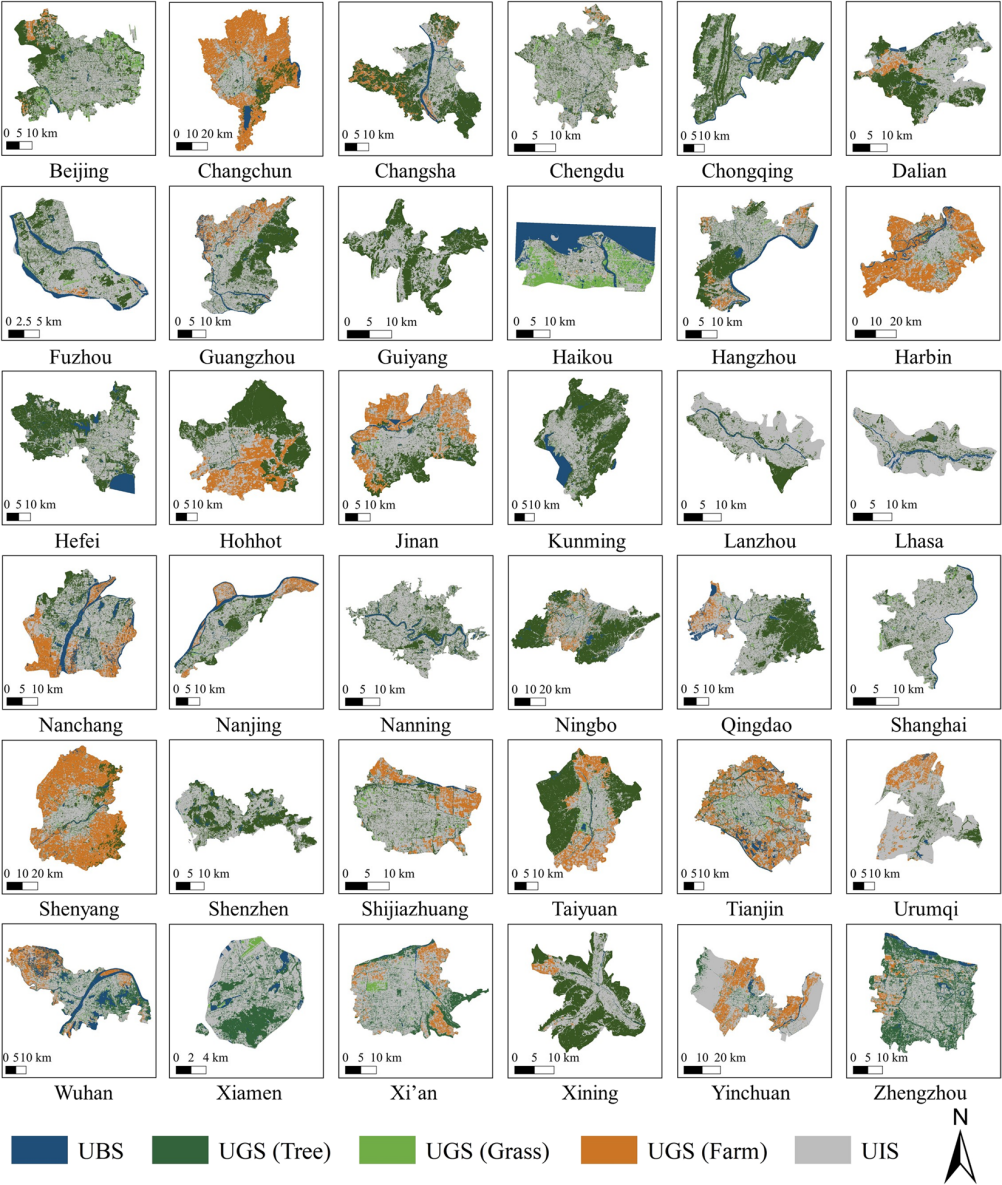
Classification maps of Urban Blue-Green-Gray Landscape dataset (UBGG-3m) for 36 Chinese metropolises.
2. **UBGGset:** the large-volume sample dataset to support the UBGG deep learning research. Complementing the UBGG-3m dataset, UBGGset serves as a large-volume sample dataset specifically tailored to support and foster UBGG research endeavors. The UBGGset consists of 14,627 sample images (without data augmentation), with dimensions of 256 pixels in length and width, covering an urban area of approximately 2,272 km^2^.

## Technical Validation

### Visual and accuracy evaluation on UBGG-3m

To evaluate the accuracy of the product, five cities were selected for visual and quantitative assessment, namely Beijing, Shenzhen, Harbin, Urumqi, and Lhasa. A total of six test sample areas were collected in these five cities, covering an area of 43.5 km^2^. The classification accuracy was evaluated by comparing the labeled reference maps with the results. The OA for all samples (about 4.83 million pixels) was found to be 91.23% (Table 3), indicating promising mapping results. For the different types, UBS had the highest F1 of 95.15%, followed by UIS at 93.14%. The F1 for trees and grass were 87.54% and 85.97%, respectively. The quantitative assessment results of different cities were accurate, with OA higher than 91% except for Lhasa (83.21%), demonstrating the usability and accuracy of the UBGG-3m product.

**Table 3:** Quantitative results of accuracy evaluation on UBGG-3m (units: %).

City	Number of pixels	UBS	UGS (Tree)	UGS (Grass)	UIS	OA	mIoU	FWIoU
		F1	F1	F1	F1			
Beijing	1364224	97.53	92.65	89.93	92.61	93.16	87.36	87.29
Harbin	693889	93.70	79.38	88.25	95.10	92.11	80.89	85.45
Shenzhen	1387778	92.01	88.14	70.94	94.52	91.49	77.14	84.51
Urumqi	693889	97.65	88.30	91.84	95.97	94.03	87.90	88.66
Lhasa	693889	92.23	76.40	56.27	82.88	83.21	64.33	71.61
Average	4833669	95.15	87.54	85.97	93.14	91.23	82.78	83.93

**Fig. 6:**
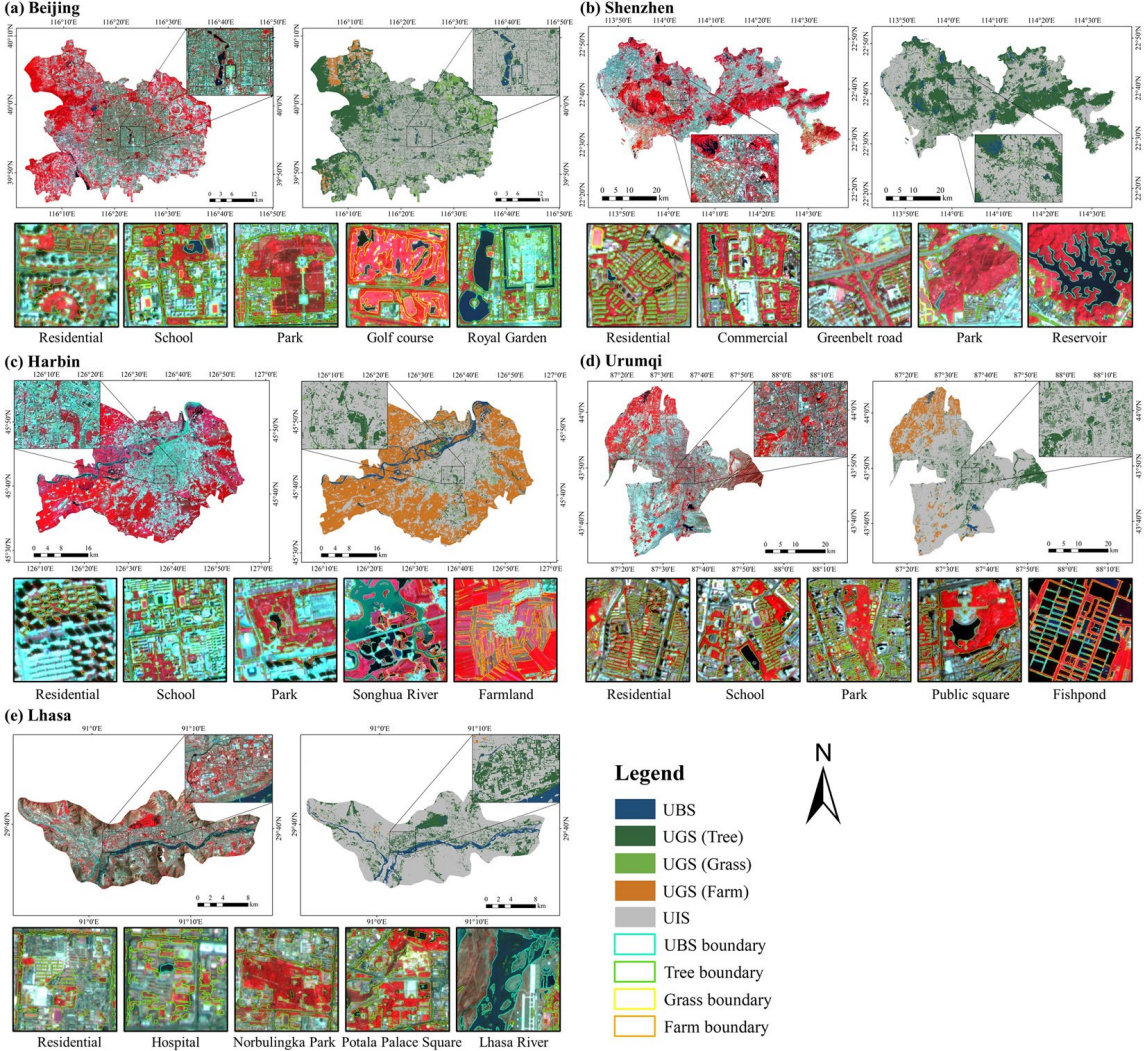
Classification results of the UBGG-3m in (**a**) Beijing, (**b**) Shenzhen, (**c**) Harbin, (**d**) Urumqi, and (**e**) Lhasa. The small maps at the bottom display detailed classification results of the UBGG landscape in major urban scenes such as residential areas, schools, parks, etc. (The background images are Planet satellite images from © Planet 2020. The classification results are depicted using colored boundaries, with bright blue representing urban blue spaces, green indicating trees, yellow representing grass, and orange denoting farmland.).

The visual assessment results of the UBGG-3m were presented in Fig. 6. As the capital of China, Beijing is a highly urbanized and economically developed city, with comprehensive blue and green infrastructure construction. The HRNet-OCR model accurately identified UBS ranging from large lakes to small ponds, as well as the moat surrounding the Forbidden City (Fig. 6a). In addition, the model also effectively captured the sizes, shapes, locations, and boundaries of UGS, such as individual tree canopies in residential areas, small arborizations in Peking University, and slender trees on boulevards. Notably, the reconstructed tree geometry was highly consistent with the ground truth data. Moreover, the model was able to successfully distinguish between trees and grass, highlighting delicate shape contours in areas such as playgrounds of a school and artificial grass on golf courses (Fig. 6a). The results demonstrated the model’s ability to extract detailed information about the UBGG landscape in urban areas and to distinguish between different types of greenery with a high level of accuracy. Shenzhen, located in the southern region of China, is characterized by a higher coverage ratio of UBS and UGS, which is mostly comprised of large reservoirs and parks. The model’s effectiveness in accurately describing the complex boundary shape of Xikeng Reservoir and identifying trees around commercial and residential buildings, as well as greenery along roadsides has been demonstrated, as shown in Fig. 6b. Harbin, as a representative city in northern China, has the largest proportion of farmland within its administrative boundaries. The analysis of detail maps indicated that building shadows had a certain impact on the accurate extraction of UGS, particularly in residential areas with tall buildings, as illustrated in Fig. 6c. The presence of building shadows led to discontinuous UGS extraction, and sometimes the obscured areas were classified as UIS, resulting in relatively poor extraction with F1 of 79.38% and 88.25% for trees and grass, respectively (Table 3). Urumqi, as a representative of inland cities in the northwest region, has the largest UIS area, and the UBGG-3m product exhibits superior performance in providing detailed information on UGS and UBS. It is worth noting that despite being geographically distant from each other, with Harbin located in the far north, Urumqi in the northwest, and Shenzhen in the south of China, the UBGG classification results for all cities are excellent. This suggests that the model framework’s performance is unlikely to be affected by geographic location differences, which could be attributed to the transfer learning strategy that helped the model adapt. Fig. 6e depicts the visualization result of Lhasa, a representative city in the Qinghai-Tibet region, where the vegetation is primarily comprised of hardy trees and alpine meadows. The growth of vegetation was affected by the phenological period, and the image of Lhasa city used in this study was taken on July 24. As the alpine meadows were still in the growing season in July, the UGS with lower and sparser vegetation cover were more likely to be misclassified, owing to their similarity in appearance to bare ground. Conversely, UGS with higher and denser vegetation cover were more accurately identified.

### Comparison with the state-of-the-art deep learning networks

**Fig. 7:**
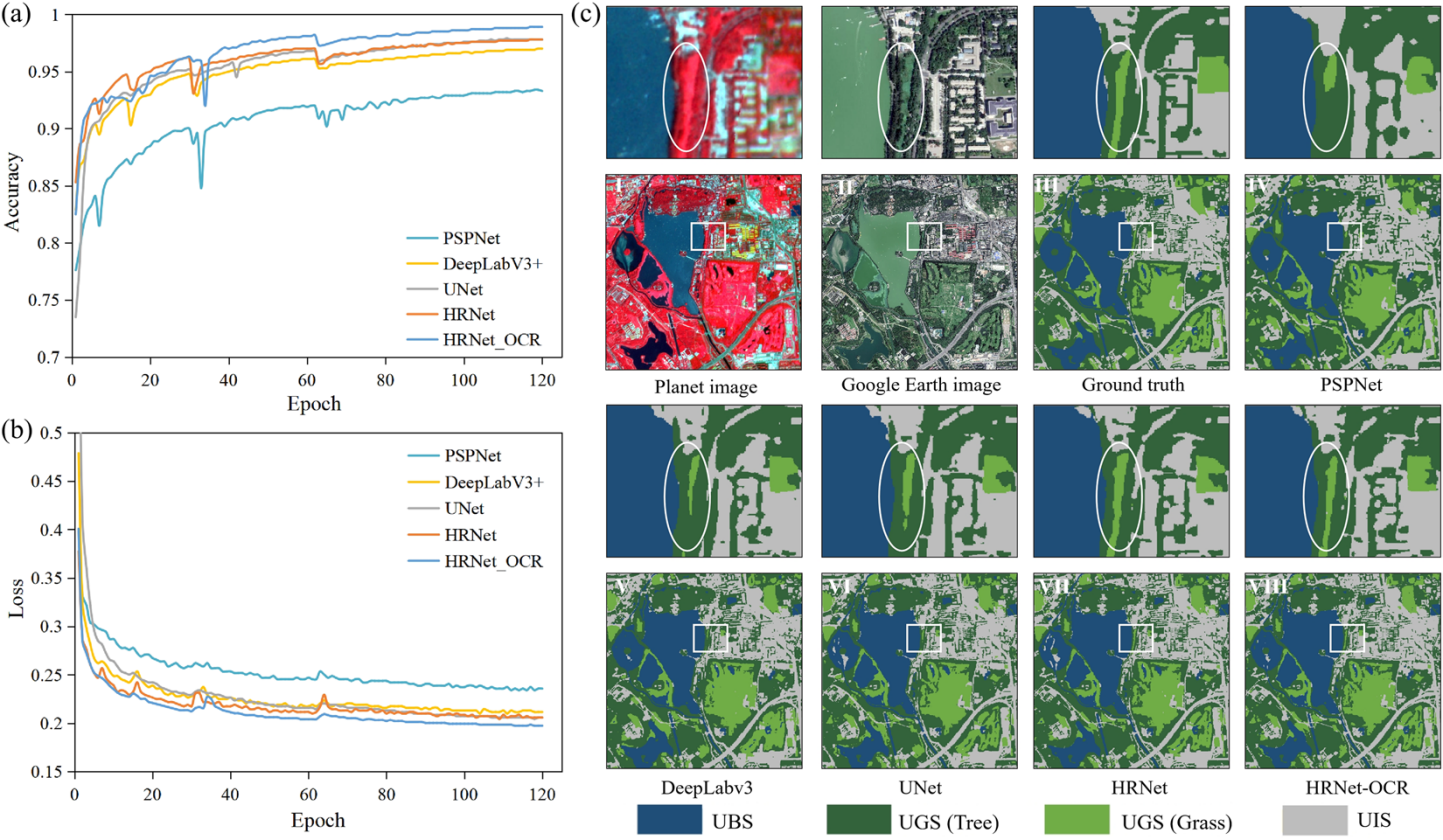
Comparison of (**a**) accuracy and (**b**) loss with epoch, and (**c**) classification results with state-of-the-art deep learning networks. The classification results include eight panels: (I) Planet satellite images from © Planet 2020, (II) Google images from © Google Earth 2020, (III) Ground truth, (IV) PSPNet, (V) DeepLabv3+, (VI) UNet, (VII) HRNet, and (VIII) HRNet-OCR.

Several state-of-the-art deep learning networks were selected for performance comparison with HRNet-OCR, including PSPNet^42^, DeepLabV3+^43^, UNet^44^, HRNet^39^. The model’s accuracy and loss value records for each round were plotted and shown in Fig. 7. As depicted in Fig. 7a, the accuracy of all five models increased rapidly with the increase in epochs and then gradually increased and stabilized after 20 epochs. In terms of training loss, as demonstrated in Fig. 7b, all five models initially showed a rapid decrease in the first 20 epochs, followed by a more stable decrease. Among the state-of-the-art deep learning networks, PSPNet exhibited the slowest improvement in classification accuracy and loss function convergence. Conversely, HRNet outperformed DeepLabv3 + in terms of accuracy improvement and loss function convergence. Overall, HRNet-OCR demonstrated the most significant training advantage, with the accuracy reaching 0.989 and the loss reduced to 0.197 after 120 epochs. Although this advantage was not apparent in the early stage, it showed significant improvement in the later stage compared to HRNet.

The performance evaluation of different models was conducted on a test region covering 1225 ha (1167 × 1167 pixels) in Haidian District, Beijing, which included Summer Palace Park, Haidian Park, Wanliu Golf Course, Kunming Lake, and Xiyuan residential area, representing a variety of UBGG landscape features (Fig. 7c). The classification results and accuracy assessment of HRNet-OCR and other state-of-the-art semantic segmentation networks are presented in Fig. 7c and Table 4, respectively. All deep learning methods demonstrated effective UBS extraction, with F1 above 96.9%. However, the classification of UGS and UIS was more challenging. PSPNet struggled to handle detailed information, resulting in smooth edges of impervious and trees that were inconsistent with the actual landscape boundaries. DeepLabv3 + still had difficulty in distinguishing trees and grass, particularly on the golf course lawns, where several solitary tree canopies were ignored. In comparison, HRNet performed better in classifying UBGG landscapes, particularly in accurately recognizing trees and grasses, with the boundaries of UGS more consistent with actual features, owing to the high to low resolution feature learning mechanism. Furthermore, the classification accuracy was significantly improved by introducing the OCR module based on HRNet. The F1 of UBS, UGS_tree, UGS_grass, and UIS classified by HRNet-OCR were improved by 0.56%, 1.11%, 1.03%, and 1.95%, respectively, compared to HRNet. The OA was ranked from large to small: HRNet-OCR (93.16%) >HRNet (91.94%) >UNet (91.05%) >DeepLabv3 + (91.00%) >PSPNet (89.40%), highlighting the effectiveness and great potential of HRNet-OCR for high-resolution landscape classification tasks.

**Table 4:** Comparison of classification accuracy with state-of-the-art semantic segmentation networks (units: %).

Methods	UBS	UGS (Tree)	UGS (Grass)	UIS	OA	mIoU	FWIoU
	F1	F1	F1	F1			
PSPNet	96.98	88.01	85.45	88.28	89.40	81.58	81.05
DeepLabv3+	97.41	89.84	86.57	90.67	91.00	83.94	83.69
UNet	96.91	90.23	86.60	90.63	91.05	83.86	83.75
HRNet	96.97	91.54	88.90	90.66	91.94	85.36	85.19
HRNet_OCR	97.53	92.65	89.93	92.61	93.16	87.36	87.29

### Comparison with and without transfer learning in large-scale UBGG landscape classification

**Fig. 8:**
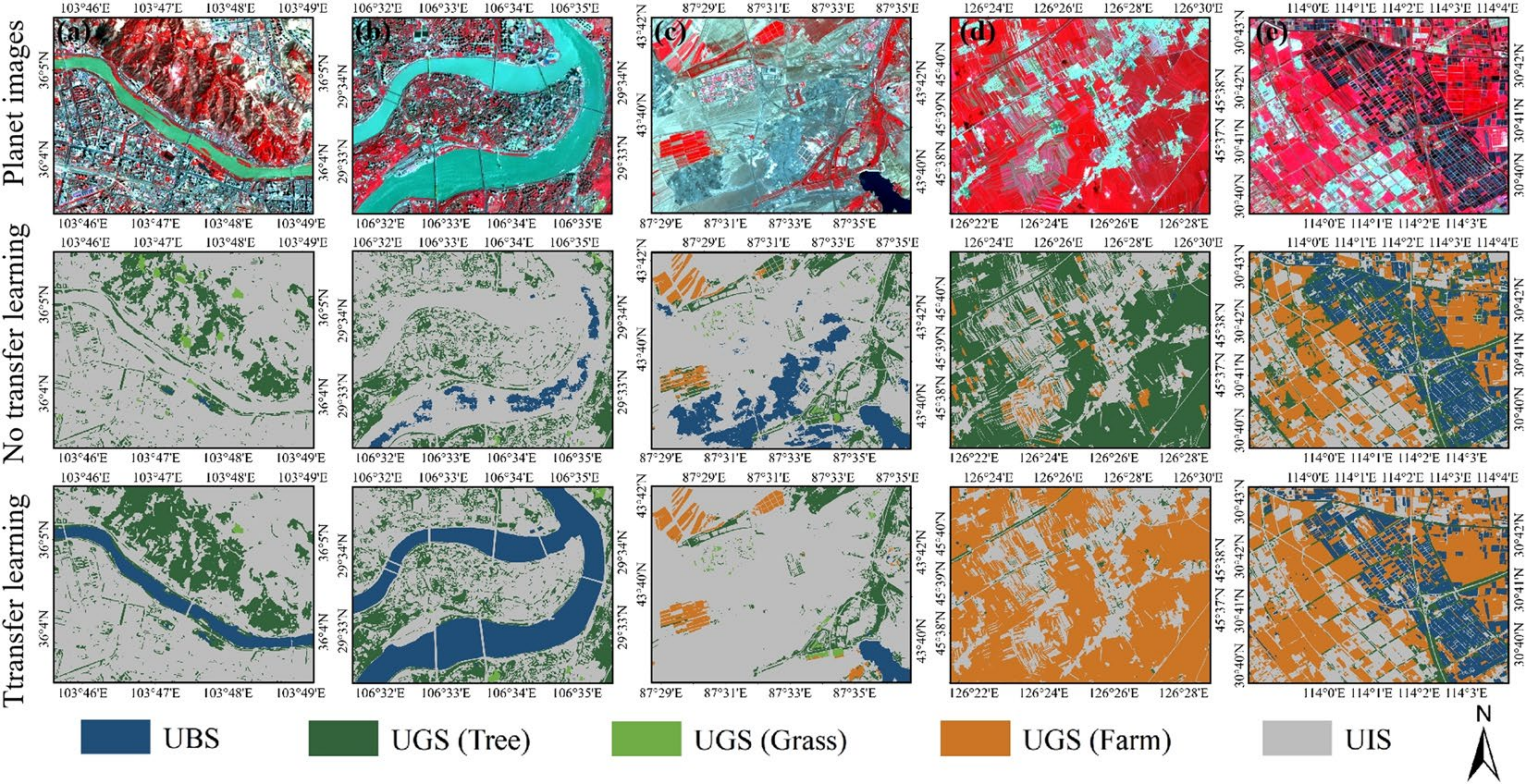
Comparison of classification results before and after transfer learning in urban landscape. (**a**) Yellow River Basin in Lanzhou; (**b**) Yangtze River and Jialing River confluence area in Chongqing; (**c**) Sand quarries in Urumqi; (**d**) Farmland in Harbin; (**e**) Aquaculture areas in Wuhan.

To develop an ecological understanding of urban systems, the spatial heterogeneity for urban landscapes from various geographic regions must be addressed for large-scale and fine-grained mapping^5,15^. Transfer learning has been demonstrated as a useful tool to address urban landscape heterogeneity and dynamics by a large body of literature^15,45^. Our study found that transfer learning can consider the spectral variance of diverse UBS types, including rivers, lakes, and reservoirs. For example, a large sediment content in the Yellow River causes a high reflectivity that appears as a blue-green color on a standard false-color image (Fig. 8a), while the Jialing River shows bright blue color due to its shallow water level, and the Yangtze River has high turbidity showing lake blue (Fig. 8b). The pre-trained model was unable to fully comprehend this UBS heterogeneity. However, after transfer learning, the misclassification was much reduced by introducing positive/negative UBS samples and fine-tuning the pre-trained model with new water features. In addition, the transfer learning cross-geographic regions method has significant advantages in solving “various UGS in the same spectrum” and “same UGS with different spectrums”^46^. For example, crops and urban trees were highly confused due to the same spectral characteristics during the peak growth period of crops in Harbin (Fig. 8d). Similarly, the classification of aquaculture area in Wuhan also had mixed trees and farmland, manifested by the relatively broken and irregular shape of farmland patches (Fig. 8e). After adversarial training, the misclassification is much improved, and the edges are more finely and accurately delineated.

The findings demonstrate that transfer learning can enhance the generalization by efficiently retraining on the pre-trained model, which is feasible and potentially possible for large-scale, high-resolution UBGG landscape mapping. In practical applications, HRNet-OCR can be automatically applied to other cities and achieve good urban landscape classification by fine-tuning the pre-trained model or even directly using the pre-trained model. Here, we counted the computational efficiency of the prediction phase. The computational times for HRNet-OCR in 36 cities were recorded based on NVIDIA Tesla P100 GPU and Pytorch. Statistically, it only took about 5 h to generate UBGG-3m covering all 36 metropolitan areas of 50, 411 km^2^ by transfer learning, which is effective for timely monitoring and managing dynamic changes in the urban landscape.

### Comparison with existing landcover/landscape datasets

**Fig. 9:**
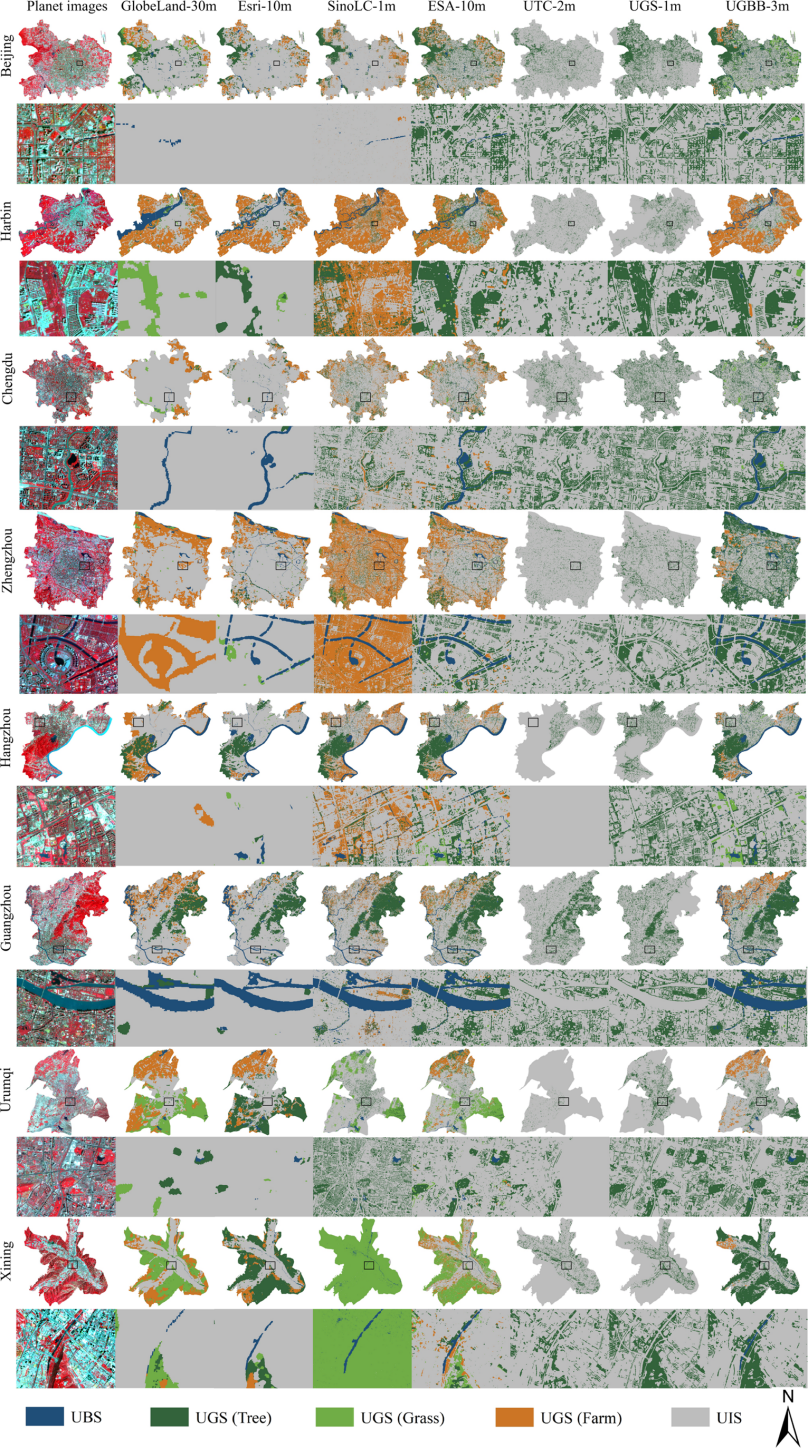
Visualization comparisons of UBGG-3m with GlobeLand 30 m^36^, Esri-10m^37^, SinoLC-1m^14^, ESA-10m^38^, UTC-2m^21^ and UGS-1m^5^.

Visualization comparisons of UBGG-3m with existing landcover/landscape datasets are shown in Fig. 9. Additionally, one region of each city was zoomed in for visual inspection of spatial detail reconstruction. Remarkably, our product demonstrated superior performance in terms of visual assessment results, exhibiting excellent landscape classification results. Most of the existing land cover products, exhibited poor accuracy in reconstructing the UBGG landscape, often misclassifying blue-green natural land as construction land (Fig. 9). Among the four large-scale land cover products, ESA World Cover displayed relatively better performance, albeit falling short in accurately depicting the edges of the urban landscape compared to UBGG-3m. This phenomenon can be attributed to two main factors. Firstly, the diameter of tree crowns typically ranges between 0.5 m and 10 m, and the width of urban rivers and ponds generally falls between 20 m to 100 m, which can be smaller than one pixel of Sentinel-2 or Landsat^21^. As a consequence, the resolution limitation leads to a mixed pixel problem, where scattered UGS and striped UBS may merge with the surrounding landscape and thus be removed from the pixel^47^. Secondly, the orientation of these products is designed for global or national land cover rather than specifically for urban areas^37^. For example, the Food and Agriculture Organization (FAO) defines forests as patches greater than 0.5 ha with more than 10% tree canopy cover, leading to an underestimation of UGS in these products.

**Fig. 10:**
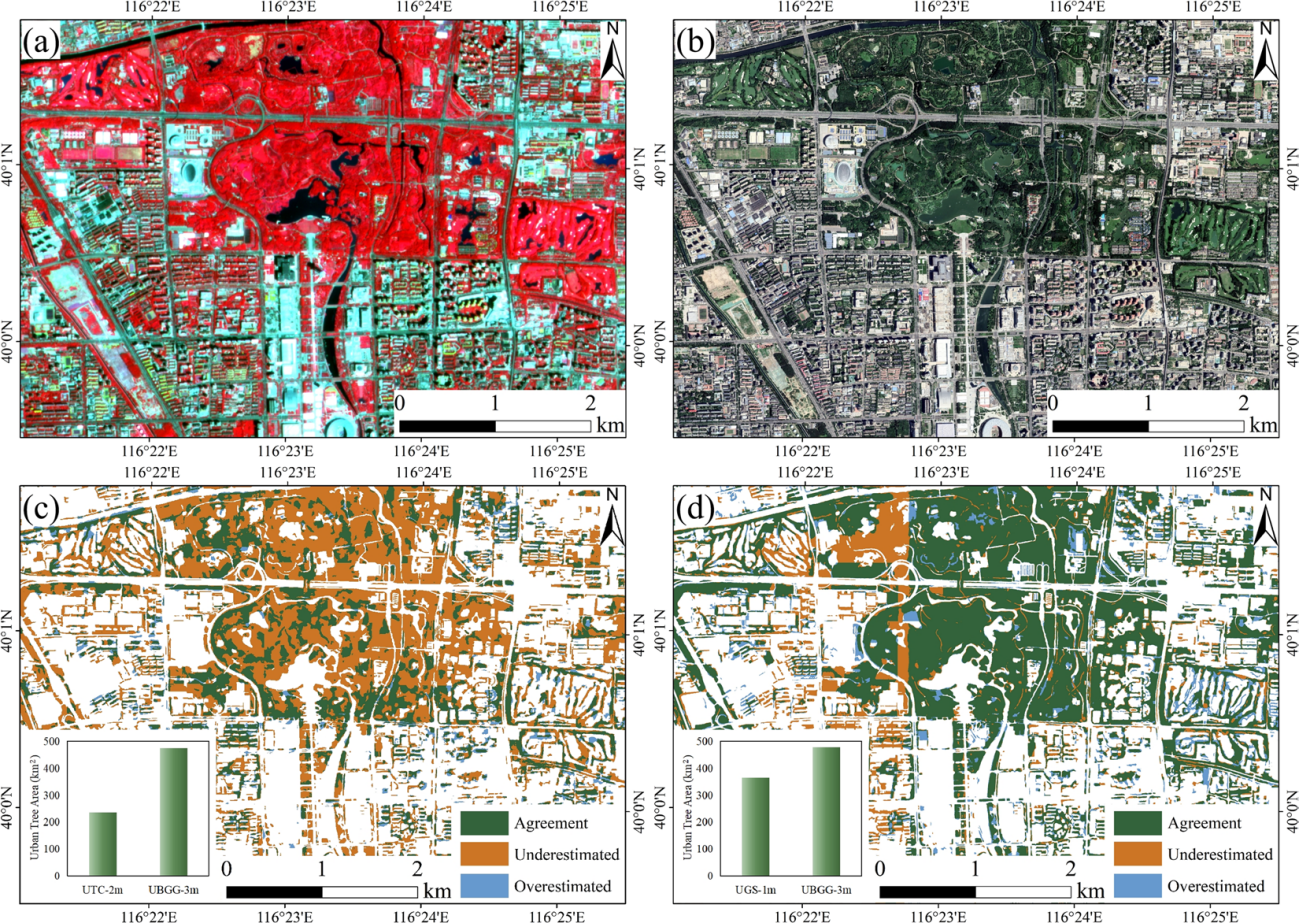
Visualization comparisons of urban tree extraction between UBGG-3m and high-resolution urban green space dataset in Beijing. (**a**) Planet satellite images from © Planet 2020; (**b**) Google Images from © Google Earth 2020; (**c**) Comparison of UBGG-3m and Urban Tree Cover-2m (UTC-2m)^21^; (**d**) Comparison of UBGG-3m and Urban Green Space-1m (UGS-1m)^5^. The green region represents the agreement between UBGG-3m and the other products in identifying urban trees. The yellow region represents the urban trees underestimated by other products compared to UBGG-3m, while the blue region represents the overestimated area by other products compared to UBGG-3m.

Furthermore, our comparative analysis with UTC-2m and UGS-1m demonstrated the superiority of our UBGG-3m product in accurately capturing urban green space (Figs. 9, 10). The UBGG-3m product based on higher resolution planet images facilitated more accurate detection of urban tree crowns and finer-grained analysis of their distribution patterns. In contrast, UTC-2m, due to its lower resolution of Sentinel-2 images, may fail to identify small or isolated trees and struggle to distinguish between different types of tree canopies. On the other hand, UGS-1m, which utilized high-resolution Google imagery, offers a comparable representation of urban green space when compared to our product. However, the utilization of multispectral information from Planet imagery allows UBGG-3m to achieve a higher level of discrimination between urban trees, grasslands, and farmlands, which is not attainable with the other two high-resolution tree products. These comparisons provide compelling evidence of the superior performance and accuracy of UBGG-3m in capturing the intricate characteristics of urban landscapes. More importantly, the UBGG-3m product mapped a comprehensive urban blue-green-gray landscape in human–nature coupled urban systems. It will enable urban planners, researchers, and policymakers to gain a deeper understanding of the complexities inherent in the urban landscape and facilitate more effective management strategies.

## Usage Notes

### Urban applications

Urban areas occupy only a very small portion of the terrestrial landscape but play a crucial role in driving environmental change at local, regional, and global scales^6,48,49^. Although the importance of urban landscape ecology is increasingly being recognized^50^, related researches are still limited due to the lack of large-scale and high-resolution urban landscape maps^29,35^. With its high resolution and accuracy, the UBGG-3m product has the potential to provide more precise knowledge of urban landscape and facilitate a deeper understanding of the patterns, processes, and implications of urbanization. Here, we briefly describe some research applications in which our product can be further applied.

1. Sustainable Urban Planning. UBGG-3m contributes significantly to the development of sustainable urban planning by providing detailed information on the spatial heterogeneity of landscape types and their distribution patterns. With the increase in urbanization, the importance of maintaining and enhancing UGS and UBS has become widely recognized^10,51^. UBGG-3m enables the identification and quantification of green and blue infrastructure, which helps in assessing their contributions to urban ecosystems and environmental services. In particular, UBGG-3m allows researchers to analyze the spatial configuration and pattern of UGS (e.g., tree, grass, and farm), including their connectivity, size, shape, type, and distribution. This information is essential for making informed decisions on urban planning and management, including land use policies, urban greening, and urban infrastructure development.
2. Urban thermal environment. Our product contributes to the in-depth study of the urban thermal environment, where the current understanding of the contributors to the Urban Heat Island (UHI) effect mainly relies on coarse land cover types due to the lack of high-resolution images^6^. However, the UHI is more like an “archipelago” than an “island”^52^, with local temperature differences as large as those along the urban–rural gradient. A systematic investigation of the interaction between fine-scale urban landscapes and thermal environments is still lacking, and UBGG-3m can provide landscapes spatial variation on a fine-scale.
3. Urban aboveground carbon storage. High-resolution urban landscape products facilitate urban aboveground carbon storage studies. Numerous studies have proven that UGS have significant carbon sink potential and provide ecosystem services and livelihood benefits^53^. However, this service has been largely underestimated in most studies. For example, an analysis conducted in Beijing showed that carbon stocks were underestimated by 39% of satellite data from 6 m to 30 m resolution^7^. Furthermore, according to an analysis in Leicester, UK^54^, shifting from 10 m to 250 m resolution remote sensing data resulted in a 76% underestimation of aboveground carbons stores. Additionally, a survey estimated that more than 1.8 billion isolated trees in West Africa have carbon stocks up to 22 MgC ha^–1^, which is far larger than global biomass mapping^23,53^. Thus, our product provides essential information on the estimation of urban aboveground vegetation carbon density with large spatial variability.
4. Deep learning. This work provides an open high-resolution dataset for urban landscape semantic segmentation studies, which can serve as a huge training pool for high-resolution land cover mapping. Moreover, Planet images cover a global scale and are freely available, allowing us to develop a robust and transferable deep network for urban landscape classification using deep learning and transfer learning. At the same time, our product also promotes more deep learning development models to be applied to urban environmental remote sensing research, driving technological advances in this field and promoting the development of urban landscape remote sensing interpretation towards intelligence and automation^17^.

Apart from the applications discussed above, the UBGG-3m can be combined with big geospatial data and contribute to other scientific research, such as smart city construction, urban digital twin, sustainability assessment, habitat evaluation, and urban health studies^29^.

### Limitations and future work

This study represents a significant advancement in the production of VHR urban landscape maps for 36 Chinese metropolises. However, several limitations of the study need to be acknowledged. Firstly, UBGG-3m only covers the 36 cities included in the study, and further work is necessary to extend this coverage to other cities worldwide. Secondly, the availability of high-resolution images is still limited by factors such as temporal resolution and cloud cover occlusion. As a result, UBGG-3m only covers the summer images of 2020-2021. As more high-resolution satellite images become available, future research could be devoted to landscape classification tasks for more cities and long time series globally. This would provide a more comprehensive understanding of urban landscape dynamics and aid in developing effective urban planning and management strategies.

## Data Availability

The programs used to generate the dataset were ENVI (5.3), ESRI ArcGIS (10.6) and Pytorch deep learning framework. All used codes to generate the dataset are available in the following GitHub (https://github.com/Zhiyu-Xu/Fine-grained-urban-blue-green-gray-landscape-dataset-for-36-Chinese-cities).
